# Characterization of the iPSC-derived conditioned medium that promotes the growth of bovine corneal endothelial cells

**DOI:** 10.7717/peerj.6734

**Published:** 2019-04-16

**Authors:** Qing Liu, Yonglong Guo, Shiwei Liu, Peiyuan Wang, Yunxia Xue, Zekai Cui, Jiansu Chen

**Affiliations:** 1 Ophthalmology Department, The People’s Hospital of Yubei District of Chongqing city, Chongqing, China; 2 Key Laboratory for Regenerative Medicine, Ministry of Education, Jinan University, Guangzhou, China; 3 Ophthalmology Department, First Affiliated Hospital of Jinan University, Guangzhou, China; 4 Institute of Ophthalmology, Medical College, Jinan University, Guangzhou, China; 5 Aier Eye Institute, Changsha, China

**Keywords:** Corneal endothelial cells (CECs), Conditioned medium, Induction pluripotent stem cells

## Abstract

Corneal endothelial cells (CECs) maintain corneal transparency and visual acuity. However, the limited proliferative capability of these cells in vitro has prompted researchers to find efficient culturing techniques for them. The aim of our study was to evaluate the use of conditioned medium (CM) obtained from induced pluripotent stem cells (iPSCs) as a source for the effective proliferation of bovine CECs (B-CECs). In our study, the proliferative ability of B-CECs was moderately enhanced when the cells were grown in 25% iPSC conditioned medium (iPSC-CM). Additionally, hexagonal cell morphology was maintained until passage 4, as opposed to the irregular and enlarged shape observed in control corneal endothelial medium (CEM). B-CECs in both the 25% iPSC-CM and CEM groups expressed and Na^+^-K^+^-ATPase. The gene expression levels of NIFK, Na^+^-K^+^-ATPase, Col4A and Col8A and the percentage of cells entering S and G2 phases were higher in the iPSC-CM group. The number of apoptotic cells also decreased in the iPSC-CM group. In comparison to the control cultures, iPSC-CM facilitated cell migration, and these cells showed better barrier functions after several passages. The mechanism of cell proliferation mediated by iPSC-CM was also investigated, and phosphorylation of Akt was observed in B-CECs after exposure to iPSC-CM and showed sustained phosphorylation induced for up to 180 min in iPSC-CM. Our findings indicate that iPSC-CM may employ PI3-kinase signaling in regulating cell cycle progression, which can lead to enhanced cellular proliferation. Effective component analysis of the CM showed that in the iPSC-CM group, the expression of activin-A was significantly increased. If activin-A is added as a supplement, it could help to maintain the morphology of the cells, similar to that of CM. Hence, we conclude that activin-A is one of the effective components of CM in promoting cell proliferation and maintaining cell morphology.

## Introduction

The corneal endothelial layer consists of a single layer of hexagonal cells that keeps the cornea transparent through corneal hydration. Corneal hydration is determined primarily by the balance between the movement of aqueous humor across the corneal endothelium into the stroma and the subsequent pumping of the fluid out from the stroma (Oshika et al., 1998). Corneal endothelial cells (CECs) retain their proliferative ability in vitro under proper culture conditions. However, they tend to display senescence or fibroblastic transformation after long-term culture in vitro (Joyce, 2012). In this study, we observed the effects of induced pluripotent stem cells conditioned medium (iPSC-CM) on CEC.

Corneal endothelial cells maintain corneal transparency, and these cells also regulate Na^+^-K^+^-ATPase pump activity and maintain barrier functions in the cornea (Okumura et al., 2016). However, human corneal endothelial cells (HCECs) have poor proliferation capacity in vivo (Joyce, 2003). There are many reasons for the loss of HCECs, including inflammation, Fuch’s endothelial dystrophy, pseudophakia bullous, keratopathy and treatments for glaucoma, and the intense loss in cell numbers cause compensatory migration and cellular enlargement (Avisar & Weinberger, 2010; Lazaro Garcia et al., 2000). When the CEC cell density is below 500 cells/mm^2^, the Na^+^-K^+^-ATPase pump and barrier functions are affected leading to irreversible corneal endothelial cell dysfunction (Nakahara et al., 2013). To date, corneal transplantation has been the most common treatment option for CEC dysfunction; however, it is accompanied by several limitations, such as surgical complications and limited donor numbers (Kinoshita, 2010). Methods have been explored to increase the proliferative capacity of these cells in vitro, such as the addition of small molecules such as Y-27632 or Y-3993 that are inhibitors of the Rho-associated coiled-coil forming kinases (ROCKs) or the addition of animal-derived extracellular matrix that promotes the adhesion and proliferation of CECs (Bednarz et al., 2000; Li et al., 2013b; Miyata et al., 2001; Okumura et al., 2012; Schönthal et al., 1999). Conditioned media can be easily collected during media changes without affecting the cultured cells. This media is rich in several small molecules and proteins that are released from the cultured cells (Pluchino & Cossetti, 2013; Ranganath et al., 2012; Ribeiro et al., 2012; Yang et al., 2013). The effects of CM have been studied extensively. CM has been used in stimulating 3D cell formation in microgravity cultures (Cazzaniga, Castiglioni & Maier, 2014). It has been shown to be useful in protecting cells against injury and promotes the regeneration and functional recovery in spinal cord and endothelial cell injury. (Bader et al., 2014; Cantinieaux et al., 2013) CM also promotes neural stem cell differentiation into neural cells (Wang et al., 2014). CM has been shown to promote hair regeneration in patients with alopecia (Fukuoka & Suga, 2015), and it can also inhibit EMT in kidney lesions (Hu et al., 2015) and support reprograming of somatic cells into stem cells (Kawamoto et al., 2018). Recent studies have found that CM promotes single iPSC proliferation by providing the conditions required for the uniform growth of these cells (Chen et al., 2012; Hu et al., 2015). Previous research has shown that CM obtained from various sources, such as embryonic stem cell-conditioned medium (ESC-CM) (Lu et al., 2010), NIH-3T3-conditioned medium, mesenchymal stem cell-conditioned medium (MSC-CM) (Kinoshita, 2010), and human amniotic epithelial cell-conditioned medium (Sha et al., 2013) has a positive role in the proliferation of HCECs. CM also helps to maintain the formation of cell monolayers that have pump activity and adherent junctions. iPSCs can differentiate into various cell types. They are similar to ESCs and are capable of self-renewal. They overcome immune rejections and the ethical questions that are often faced by ESCs (Kotton, 2012; Lundin et al., 2018; Ma et al., 2017; Sontag et al., 2017).

In our study, we provide evidence suggesting that CM obtained from iPSCs can stimulate the proliferation and motility of bovine corneal endothelial cells (B-CECs) while maintaining the formation of a contact inhibited monolayer with functional adherent junctions and pump functions. Our findings suggest that iPSC-CM may employ PI3-kinase signaling in regulating cell cycle progression, which can cause the proliferation of B-CECs. We were also able to prove that activin A plays a key role in the proliferation of the cells. Although B-CECs are relatively easy to obtain and have been used for in vitro CEC studies, the proliferative ability of these cells is limited and is similar to that of HCECs in culture. Human tissue sources are limited and difficult to obtain; hence, the investigation of cellular behaviors in B-CEC models maybe extrapolated to human CECs. Our study provides insights into future implications of CEC treatment options.

## Materials and Methods

### Ethics statement

The bovine eyes were obtained at a local slaughterhouse (Jiang Village, Duo Ying Poultry Co. Ltd., Guangzhou, Guangdong, China), and the tissues were obtained in accordance with the association for research in vision and ophthalmology (ARVO) statement on the use of animals in ophthalmic and visual Research.

### Materials

Dulbecco’s modified Eagle’s medium (DMEM), fetal bovine serum (FBS), penicillin-streptomycin and trypsin (0.25%) and ethylenediaminetetraacetic acid (EDTA) (0.02%) were purchased from Invitrogen-Gibco (Grand Island, NY, USA). mTeSR1 was purchased from STEMCELL Technologies (Vancouver, BC, Canada), cell counting kit-8 (CCK-8) was purchased from Dojindo (Kyushu, Japan), Annexin-V-FITC/PI apoptosis detection kits were purchased from KeyGEN (Nanjing, China), and a flow cytometry (BD, Franklin lake, NJ, USA) was used. The live-dead cell count kit was purchased from Biotium (Fremont, CA, USA). Goat anti-rabbit IgG secondary antibody, rabbit polyclonal anti-AQP1 antibody, and rabbit polyclonal anti-Na^+^-K^+^-ATPase antibody were purchased from Santa Cruz Biotechnology (Santa Cruz, CA, USA). Rabbit polyclonal anti-GAPDH (1:3,000) was from Abcam (Cambridge, UK), and rabbit polyclonal anti-phosphorylated Akt (1:2,000) was from Cell Signaling Technology (Danvers, MA, USA). Y-27632 (ROCK inhibitor), Matrigel and fluorescein sodium salt were obtained from Sigma–Aldrich (St. Louis, MO, USA). The EZgene™ Tissue RNA Miniprep Kit was from Biomiga (San Diego, CA, USA). The ReverTra Ace qPCR RT Kit and SYBRH Premix Ex Taq™ Kit were from Toyobo (Osaka, Japan). Primers were synthesized by BGI (Beijing, China). A real-time PCR detection system (Bio-Rad, Hercules, CA, USA) was used for q-PCR experiments; RIPA buffer containing a protease inhibitor cocktail was purchased from Beyotime Biotechnology (Shanghai, China). Additional materials included a BCA™ Protein Assay Kit (Takara Bio, Kusatsu, Japan), PVDF membranes (Bio-Rad, Hercules, CA, USA), nonfat dry milk (Cell Signaling Technology, Danvers, MA, USA), an enhanced chemiluminescence (ECL) Advance Western Blotting Detection Kit (GE Healthcare, Piscataway, NJ, USA), immunostaining band (Tanon2500), 0.22-µm filtration unit (EMD Millipore Corporation, Billerica, MA, USA), six-well and 96-well plates (Corning, Corning, NY, USA), and transwell inserts (0.4 μm; BD, Franklin Lake, NJ, USA), human Activin A Quantikine ELISA Kit (BD, Franklin lake, New Jersey, USA).

### Isolation and culture of B-CECs from bovine corneal tissue

Bovine eyes were obtained in accordance with the ARVO statement on the use of animals in research. The eyes were purchased from a local slaughterhouse within 6 h after death. We isolated and cultivated the B-CECs as described previously, with some modifications (Satpathy et al., 2004). In detail, the eyeballs were covered with ice-cold phosphate-buffered saline (PBS) containing 2% penicillin-streptomycin and 50 μg/ml gentamicin for 30 min. The cornea was dissected from the eyeball, with a one to two mm sclera rim left. The posterior surface of the corneal tissue was then digested with trypsin (0.25%)-EDTA (0.02%) at 37 °C for 0.5–1 min, and the endothelial cells were obtained by gently scratching the Descemet’s membrane using a surgical micro spatula while viewing under a dissecting microscope. The cells were then centrifuged at 1,500 rpm for 5 min and were seeded at a concentration of 1 × 10^4^ cells per well into a six-well culture plate. They were then incubated at 37 °C and 5% CO_2_ in a humidified cell culture incubator with CEM containing low-glucose DMEM supplemented with 10% FBS and 1% penicillin-streptomycin. The culture medium was replaced every 2 days. After the B-CECs grew into a confluent monolayer (5–7 days after plating), the cells from passages 1–4 were used in all experiments.

### Preparation of iPSC-CM

We used the UMC human iPSCs line in all experiments, which was generated from umbilical cord matrix and amniotic membrane mesenchymal cells by transduction of retroviral factors, including *OCT4*, *SOX2*, *C-MYC*, and *KLF4* (Cai et al., 2010). We cultivated the iPSCs as previously described (Zhao et al., 2012). Briefly, iPSCs were cultured at 37 °C and 5% CO_2_ in a humidified cell culture incubator with mTeSR1 medium. The culture plates were precoated with 1% Matrigel before cell seeding. The cell medium was changed daily, and the changed medium was pooled and centrifuged at 1,250 rpm for 5 min. The supernatant was filtered through a 0.22-μm filtration unit to remove dead cells. The collected medium was preserved at −80 °C for at least 1 week. The addition of a certain percentage of conditioned medium into the bovine corneal endothelium medium (CEM) generated the iPSC-CM medium. iPSC cells were passaged every 6 days, and ROCK inhibitor Y-27632 (10 mM) was added to each well on the first day after each passage.

### Optimization of iPSC-CM concentration

To compare the optimum proliferation ability between the CEM group and the iPSC-CM group, we seeded the first passage of B-CECs at the same cell density of 1 × 10^3^ cells/well into 96-well culture plates. The cells were then cultured in two different mediums: CEM containing fresh iPSC medium (mTeSR1 medium) at concentrations of 0%, 5%, 25%, and 50%, and CEM containing iPSC-CM at concentrations of 5%, 25%, and 50%. After 24 h, the proliferation ability was evaluated by CCK-8 assay, as previously described (Dai et al., 2012). Briefly, 10 µl of CCK-8 solution was added to each well and the cells were incubated in the dark at 37 °C for 2 h. Next, a multimode reader was used to measure the absorbance of each well at 450 nm. Each group contained six different wells per plate to assess the cell proliferation.

### Live cell count assay and morphology changes

Primary cells in the exponential growth phase were apportioned into six-well culture plates at a density of 1 × 10^4^ cells/well in two mediums: CEM (control group) or iPSC-CM (experimental group) at the optimized concentration. A live cell count assay (*n* = 3) was performed using a live/dead cell count kit. The assay shows green fluorescence of calcein acetoxymethyl ester (calcein AM) stain in live cells and red fluorescence of ethidium homodimer III stain in dead and damaged cells. After 1, 3, and 5 days the samples were incubated with working solutions of live/dead stain (two μM calcein AM in PBS). The samples were then washed thrice with PBS and photographed using a fluorescence microscope. Each group contained three samples, and the average number of live cells was counted using the ImageJ software. The number of live cells obtained was then used to plot a cell proliferation curve. To observe morphology changes, 1 × 10^4^ B-CECs were maintained in CEM or iPSC-CM for 28 days in one passage, and the B-CECs were passaged every 7 days. Phase-contrast microscopy was used to record the morphology changes of each group.

### Real-time quantitative polymerase chain reaction analysis

Bovine corneal endothelial cells were seeded at a cell density of 1 × 10^5^ cells/well into six-well plates with either CEM or iPSC-CM. Total RNA was isolated after 7 days using the EZgene™ Tissue RNA Miniprep Kit, according to manufacturer’s protocol. RNA samples were quantified by measuring the OD at 260 nm. The 260/280 OD ratios for all RNA samples were between 1.8 and 2.1. Total RNA (one µg) was reverse transcribed to cDNA using the ReverTra Ace® qPCR RT Master Mix. The cDNA was synthesized using the CFX96 real-time PCR detection system. The reaction mixture consisted of 12.5 μl of SYBRH Premix Ex Taq™ (2×), 0.5 μl of forward and reverse primers (10 mM), two μl of diluted cDNA and 9.5 μl of ddH_2_O. Primer sequences are shown in Table 1. The reaction protocol was 95 °C for 30 s, followed by 40 cycles of 95 °C for 5 s, and 58 °C for 30 s. The relative expression levels of Na^+^-K^+^-ATPase, Col4A, Col8A, and ki-67 were normalized against β-actin. The melting curves were examined for the quality of PCR amplification of each sample, and quantification was performed using the comparative CT (2^−ΔΔct^) method.

**Table 1:** List of primers.

Gene name	Primer sequence (5′ to 3′)		Product (bp)
Na^+^/K^+^- ATPase	Forward	CAGAAATCCCAAAACAGACAAAC	243
	Reverse	TCTCAGCCAGAATCACAAAGTAA	
Col4A	Forward	CAACAGAGGACTTGGTTTCTACGGA	137
	Reverse	TGTACTGATCTGGGTGGAAGGTGA	
Col8A	Forward	GGAGTAGGTAAGCCAGGAGTGACAG	138
	Reverse	AATTCCAGGTATCCCAGGAGGTC	
NIFK	Forward	ATGTTAAATTAGTTTGTGGTGGGCT	110
	Reverse	TCAGGTGGTCACTACATGAATCTTG	
β-actin	Forward	CCGCCGCCGATGATTGTTA	142
	Reverse	CCGCCGCCGATGATTGTTA	

### Immunofluorescence assay

Immunofluorescence was used to examine the protein expression of AQP1 and Na^+^-K^+^-ATPase treated with iPSC-CM. Cells were seeded as in the RT-PCR experiments. The experimental group and the control group were treated with iPSC-CM and CEM, respectively, for 7 days. Briefly, after the cells were confluent, they were washed with PBS three times and fixed in 4% paraformaldehyde for 30 min at room temperature. Next, the cells were washed again with PBS and incubated with PBS containing 5% FBS and 0.1% Triton X-100 for 30 min at room temperature. The cells were then incubated with the primary antibodies rabbit polyclonal anti-AQP-1 (1:200) and rat polyclonal anti-Na^+^-K^+^-ATPase (1:200) overnight at 4 °C, followed by incubation with secondary antibodies for 60 min before staining with DAPI. The cells were examined by fluorescence microscopy.

### Flow cytometry analysis

Passage 1 cells at a density of 1 × 10^4^ cells/well were treated with iPSC-CM or CEM for 5 days and harvested using 0.25% trypsin-EDTA. They were then centrifuged at 1,000 rpm for 5 min. The cell pellets were suspended in 300 μl of PBS containing 2% FBS and fixed with 700 µl of cold 70% absolute ethanol for 1 h at 4 °C. The cells were then centrifuged at 2,000 rpm/min for 10 min and washed with 500 µl of PBS. This was followed by centrifugation at 200 rpm for 10 min, and the pellets were then suspended in PI and incubated in the dark for 30 min at 4 °C. The cell cycle was analyzed by flow cytometry, and each group contained three independent samples. Apoptosis was analyzed by flow cytometry using an Annexin-V-FITC/PI Apoptosis Detection Kit. We selected B-CECs in the 4th passage as experimental cells, and the B-CECs were cultured as in the cell cycle analysis experiments, with iPSC-CM or with CEM, until the cells were approximately 60–70% confluent (2–3 days after plating). The cells were harvested and stained according to the manufacturer’s instructions. In detail, the cells were centrifuged at 1,000 r/min for 5 min, resuspended in 200 µl of PBS with the addition of two µl of Hoechst and two µl of PI, followed by incubation in the dark for 15 min at 37 °C. The samples were then immediately analyzed by flow cytometry. The data analysis was performed by FlowJo software and take single gate, analyze the apoptotic histogram and cycle histogram to obtain apoptosis and cycle information. The results were expressed as the mean of the percentage of three independent experiments.

### Scratch wound healing assay

The method was performed as previously described (Li et al., 2013b). Confluent B-CECs were mechanically wounded with a one ml plastic pipette tip to create a uniform wound, and then the medium was changed into either iPSC-CM (experimental) or CEM (control). Wound closure was recorded at 0, 9, and 24 h using a phase-contrast microscope. Each group contained three samples, and ImageJ software was used to calculate the wound areas.

### Fluorescein leakage test

The fluorescein leakage test (FLT) is performed as previously reported (Guo et al., 2015). Cells were seeded at a density of 1 × 10^5^ cells/well on the inside of the 0.4 μm pore size of 24-well transwell insert, either in CEM or in iPSC-CM. The FLT test was carried out at days 3, 5, and 7 after cell seeding. The cells were washed three times with PBS before the addition of 0.5 ml of 10 μg/ml fluorescein sodium to the inside of the insert at 37 °C for 30 min. The inserts were then removed and the amount of fluorescein in each well was determined on a multimode reader, measuring the absorbance of each well at 450 nm. Each group contained six different wells per plate to assess the barrier function of the cell. An insert without cells was used as a blank control, and the fluorescein leakage of all experimental groups was compared with the blank control group.

### Western blot analysis

Confluent cells in the same passage were starved by culturing in a medium without serum overnight, followed by changing the medium to CEM or iPSC-CM for 30 min and 180 min. The cells were then lysed with cold RIPA buffer containing a protease inhibitor cocktail and were sonicated on ice. After 20 min, the sonicated material was collected and centrifuged for 20 min at 15,000 r/min at 4 °C. The supernatant was collected, and the protein concentration of the sample was assessed by using the BCA™ Protein Assay Kit. Proteins of the same molecular weight were separated on 10% SDS-PAGE gels and transferred onto a PVDF membrane. Subsequently, the membranes were blocked with 3% nonfat milk in TBS-T buffer (50 mM Tris, pH 7.5, 150 mM NaCl_2_, and 0.1% Tween-20) for 1 h at room temperature, followed by overnight incubation at 4 °C with the following primary antibodies: GAPDH (1:3,000) and phosphorylated Akt S473 (1:2,000). Next, the blots were washed and then incubated with goat anti-rabbit IgG secondary antibody (1:3,000) for 1 h at room temperature. The blots were then washed again and developed with luminal for ECL using the ECL Advance Western Blotting Detection Kit. The quantitation of band intensities was performed by scanning the immunostained band (Tanon2500; Tanon Technology Co., Ltd., Shanghai China) and analyzing the image with ImageJ software. Each group contained three samples.

### Conditioned medium effective component analysis

#### ELISA

We used commercially available enzyme-linked immunosorbent assay kits to test the levels of activin A in CEM and iPSC-CM. The protocol was followed as per the manufacturer’s instructions. Briefly, the standard solution of activin A provided in the kit along with CEM and iPSC-CEM was added into the provided microwells, followed by the addition of 100 µl of combined enzyme and incubated for 30 min at 37 °C. After incubation, the wells were washed five times and dried by gentle tapping. Next, 100 μl of the respective substrate buffers were added, and the mixture was allowed to react in the dark for 30 min, followed by the addition of 50 μl of termination solution. The absorbance at 450 nm was measured for each well.

#### Morphological observation

Bovine corneal endothelial cells at passage 4 were seeded at a density of 1 × 10^4^ cells/well in six-well culture plates. The cells were cultured using CEM, iPSC-CM, or activin A medium. The cell morphology was observed after a week under an inverted microscope. Each group contained three samples.

#### Statistical analysis

Each experiment contained three to six samples. The statistical significance (*P*-value) of two sample mean value comparisons was determined by Student’s *t*-test, and comparisons of multiple samples were analyzed by one-way ANOVA. The results were expressed as the mean ± SEM.

## Results

### Optimization of the concentration of iPSC-CM

**Figure 1:**
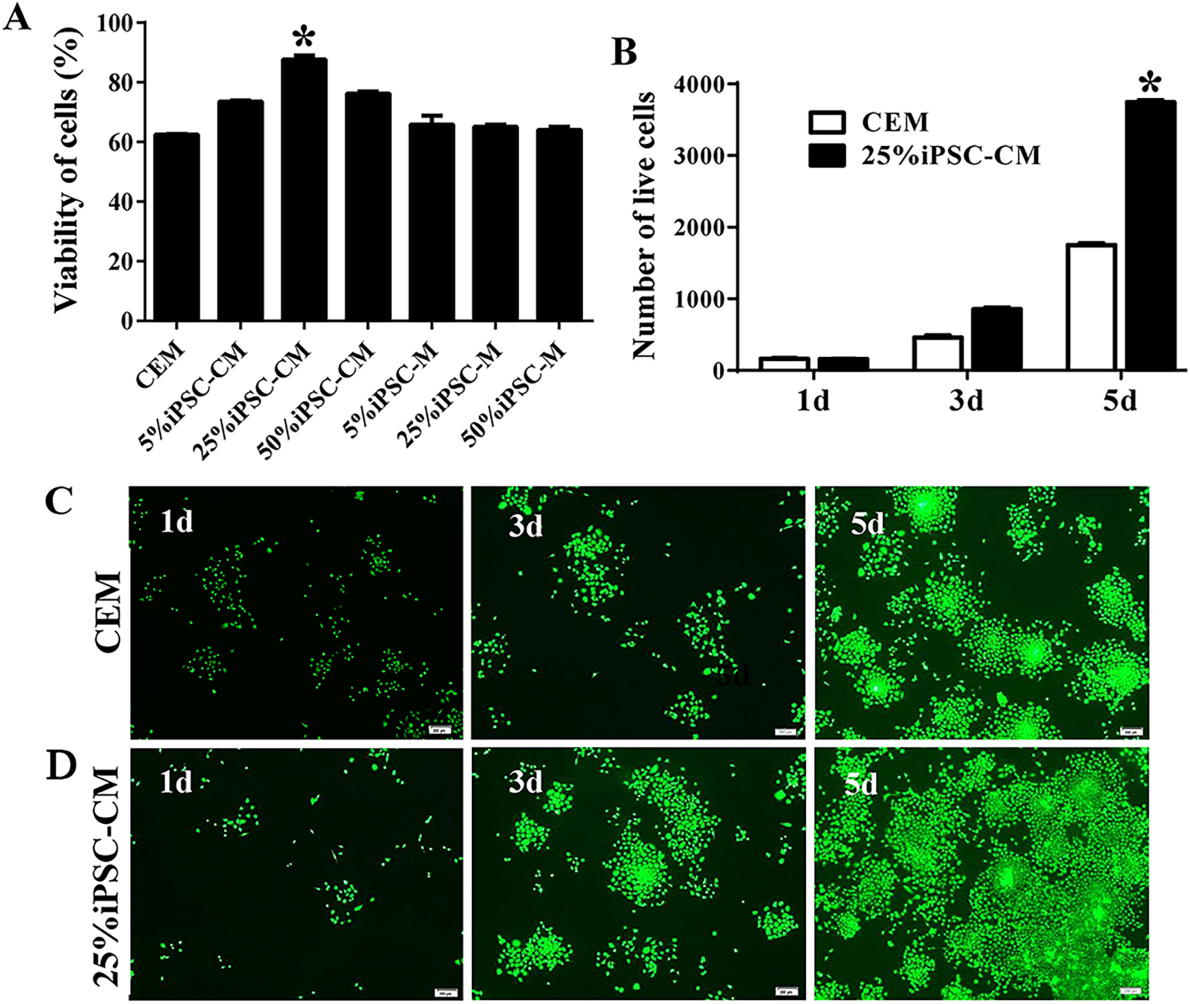
The proliferation of B-CECs treated with iPSC-CM was detected by CCK-8 analysis and live cell stain. (A) Cell number in three different mediums. The B-CECs were seeded at a density of 1 × 10^3^ cells/well in 96-well plates. The CEM, iPSC-CM, and iPSC-M medium were added to passage 1 cells. The data are expressed as the mean ± SEM (*n* = 6). The results showed that the 25% iPSC-CM group had the best proliferation and had statistically significant differences to the other groups (*P < 0.05 was considered statistically significant; versus CEM group, *n* = 6). (B) The live cell count of the difference in the 25% iPSC-CM group was statistically significant (*P < 0.01 was considered significantly significant, versus CEM group, *n* = 3). Primary B-CECs live cell stain in CEM (C) and in 25% iPSC-CM (D) under phase-contrast microscopy. Scale bar: 200 μm.

A CCK-8 was used to assay the viability of B-CECs with conditioned medium or CEM treatments. Compared with CEM (as shown in Fig. 1A), the iPSC-CM moderately promoted the proliferation of B-CECs (*P* < 0.05, *n* = 6). The condition of 25% iPSC-CM showed the largest proliferation, while the number of B-CECs decreased upon incubation with higher concentrations (50%) of iPSC-CM. Fresh iPSC medium (mTeSR1 medium) at the concentrations of 5%, 25%, and 50% showed no significant difference to CEM (*P* > 0.05, *n* = 6). The assay demonstrates that the 25% concentration was optimal for iPSC-CM to enhance the proliferation of B-CECs in vitro. Based on these results, we used 25% iPSC-CM as the fixed concentration to treat B-CECs, and we can conclude that iPSC-CM, rather than fresh iPSC medium, promotes B-CEC proliferation.

### The maintenance of CEC phenotype after treatment with iPSC-CM

**Figure 2:**
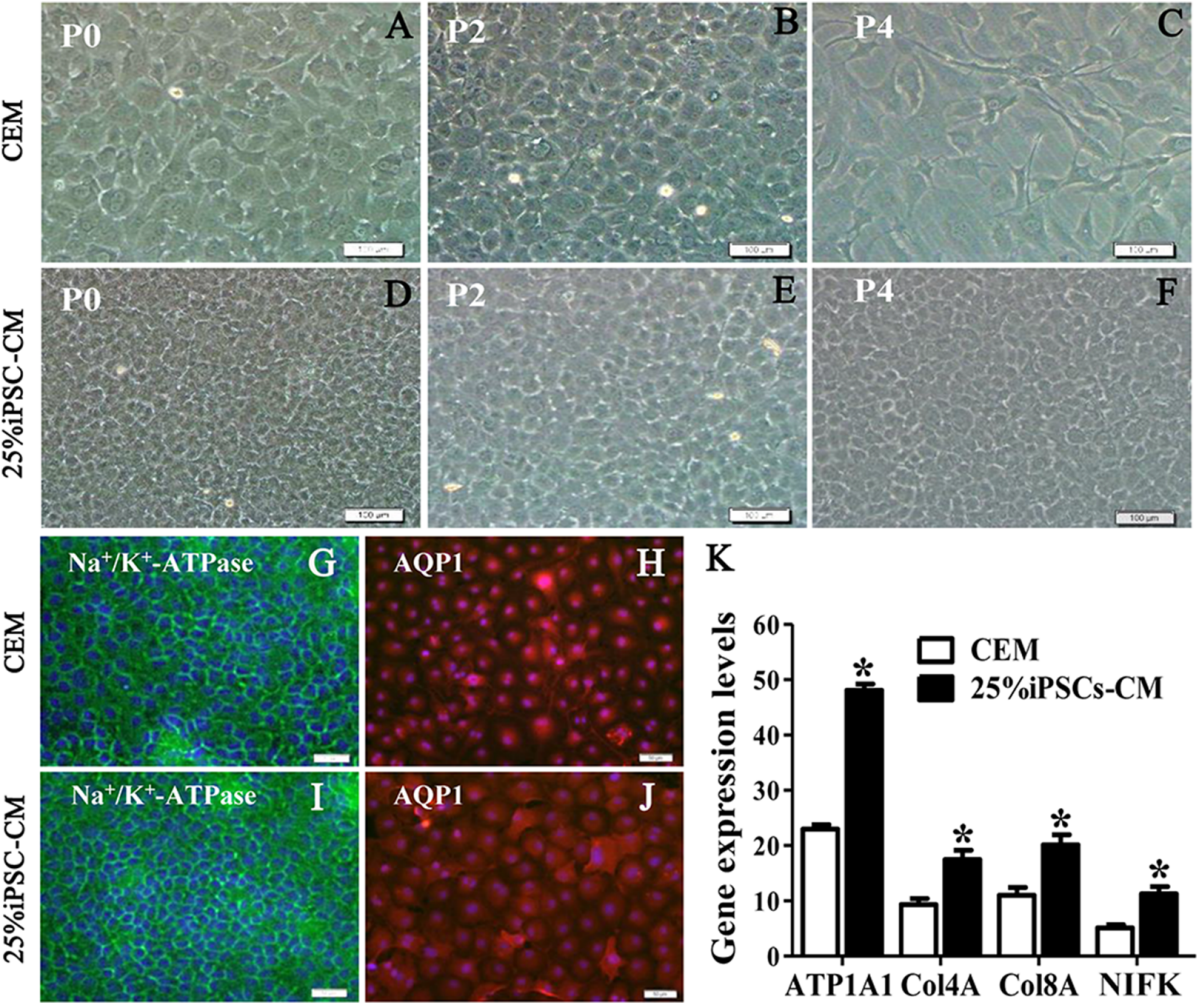
The maintenance of CEC phenotype after treatment with iPSC-CM. Morphological change of B-CECs in P0 (A, D), P2 (B, E), and P4 (C, F) under phase-contrast microscopy. Scale bar: 200 μm. (G–J) The protein expression of Na^+^-K^+^-ATPase and AQP1 by immunocytochemistry. Scale bar: 100 μm. Immunofluorescence staining showed that the 25% iPSC-CM group and the CEM group both expressed the same endothelial marker proteins, Na+/K+-ATPase, and AQP1. (K) The mRNA expression of ATP1A1, Col4A, Col8A, and NIFK by qPCR. The qPCR showed that the 25% iPSC-CM group had a higher expression than the CEM group, both in the endothelial marker genes ATP1A1, Col4A, Col8A and in the proliferation-related gene NIFK, and the difference was statistically significant (*P < 0.05 was considered statistically significant; versus CEM group, *n* = 3). Scale bar: (A) 100 μm; (B) 50 μm.

The growth kinetics tests exhibited that more live cells (green fluorescence) grew in the iPSC-CM group than in the CEM group, and there was a significant difference between the two groups (*P* < 0.01, *n* = 3), as shown in Figs. 1B and 1C. B-CECs at P0 to P2 in the CEM group and the iPSC-CM group both showed endothelial morphology, whereas cells at passage 4 maintained in CEM (Figs. 2A–2E) were larger in size and lost the native hexagonal cell morphology. Cells in iPSC-CM (Fig. 2F) still displayed a monolayer of hexagonal cell morphology and demonstrated contact inhibitions. Immunostaining of AQP1 and Na^+^-K^+^-ATPase was clearly outlined in B-CECs maintained with either CEM (Figs. 2G–2H) or iPSC-CM (Figs. 2I–2J). These data indicated that iPSC-CM apparently did not alter the cell-type specificity of B-CECS. The qPCR data showed that the relative expression levels of the functional genes Na^+^-K^+^-ATPase, COL4A, COL8A, and proliferation-related gene NIFK in the B-CECs at passage 2 from the iPSC-CM group were higher than those in the CEM group, and this was statistically significant (*P* < 0.05, *n* = 3), as shown in Fig. 2K. According to the results, we conclude that iPSC-CM can not only maintain the corneal endothelial phenotype but also improve corneal endothelial cell growth and proliferation.

### The promoting effect of iPSC-CM on the survival of B-CECs

**Figure 3:**
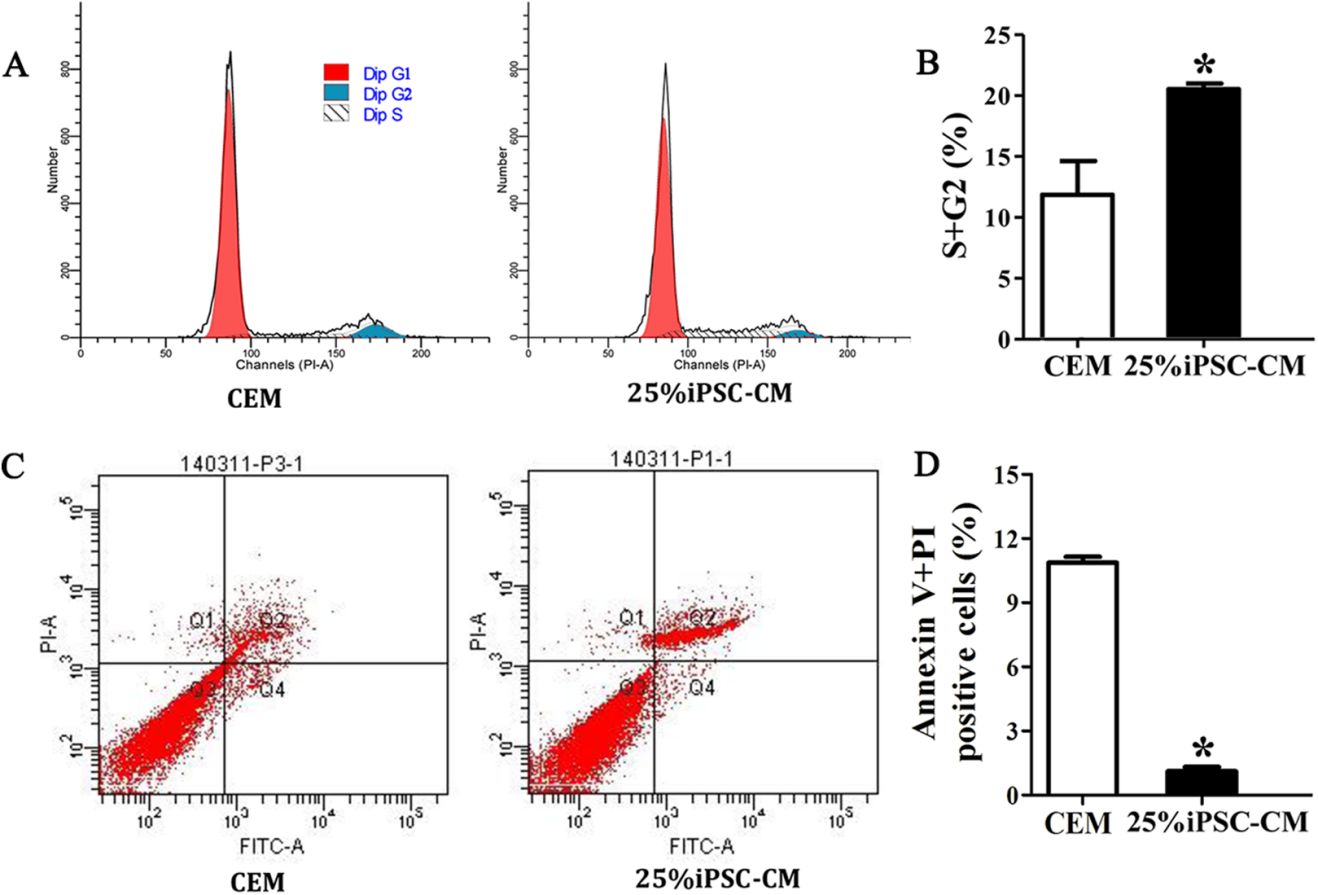
iPSC-CM treatment promoted antiapoptotic activity in cultured B-CECs. (A) Cell cycle analysis of B-CECs between the 25% iPSC-CM group and CEM group. (B) Quantification of the cell cycle assays showed the percentage of cells entering the S and G2 phases were higher in 25% iPSC-CM than in CEM. (C) Annexin V and PI assay of the B-CECs in the 25% iPSC-CM group and CEM group. The Q1 area represents cell necrosis; Q2 represents late-apoptotic cells; Q3 represents viable cells; and Q4 represents early apoptotic cells. (D) Quantification of the Annexin V and PI assays. The percentage of apoptotic cells was calculated from Q2 and Q4. The number of apoptotic cells decreased in the 25% iPSC-CM group, which showed a statistically significant difference (*P < 0.05 was considered statistically significant; versus CEM group, *n* = 3).

**Figure 4:**
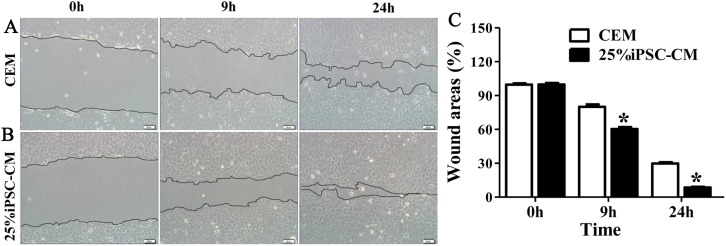
Effect of iPSC-CM on the migration of B-CECs. Wound healing was shown for cultured B-CECs in the 25% iPSC-CM group (B) and CEM group (A) (at 0, 9, and 24 h) under phase-contrast microscopy. (C) The percentage of wound areas were determined with ImageJ analysis software. A *P*-value of <0.05 was considered to be statistically significant (*P < 0.05 versus CEM group). Scale bar: 100 μm.

Flow cytometry analysis showed that the cell-cycle process of B-CECs in iPSC-CM was dramatically promoted, as shown in Figs. 3A and 3B. The percentage of B-CECs entering the S phase and G2 phase in the iPSC-CM group and CEM group were 20.54% ± 3.45% and 11.87% + 2.77%, respectively. An Annexin V/PI kit was used to detect apoptotic and necrotic CECs at P4. The flow cytometry results revealed that 25% iPSC-CM treatment significantly decreased apoptosis (*P* = 0.001, *n* = 3) (Figs. 3B and 3C). The number of apoptotic and necrotic cells was 10.87% ± 0.46% and 1.10% ± 0.36% in the CEM and iPSC-CM groups, respectively. In summary, these results prove that iPSC-CM can not only promote the proliferation of B-CECs but also prevent the apoptosis of B-CECs. The effect of iPSC-CM on B-CEC scratch-induced migration was evaluated by scratch experiments. The wound margins were blurry after 9 h of scratch, and the wound area was approximately 80.10% ± 3.60% in the CEM group and 60.36% ± 2.90% in the iPSC-CM group. After 24 h, in the CEM, most of the wound areas were covered by B-CECs (29.76% ± 1.92%), and the wound areas were almost closed in the iPSC-CM (8.60% ± 0.91%) (Figs. 4A–4C, *P* < 0.05, *n* = 3).

### The effect of iPSC-CM on the barrier function of B-CECs

**Figure 5:**
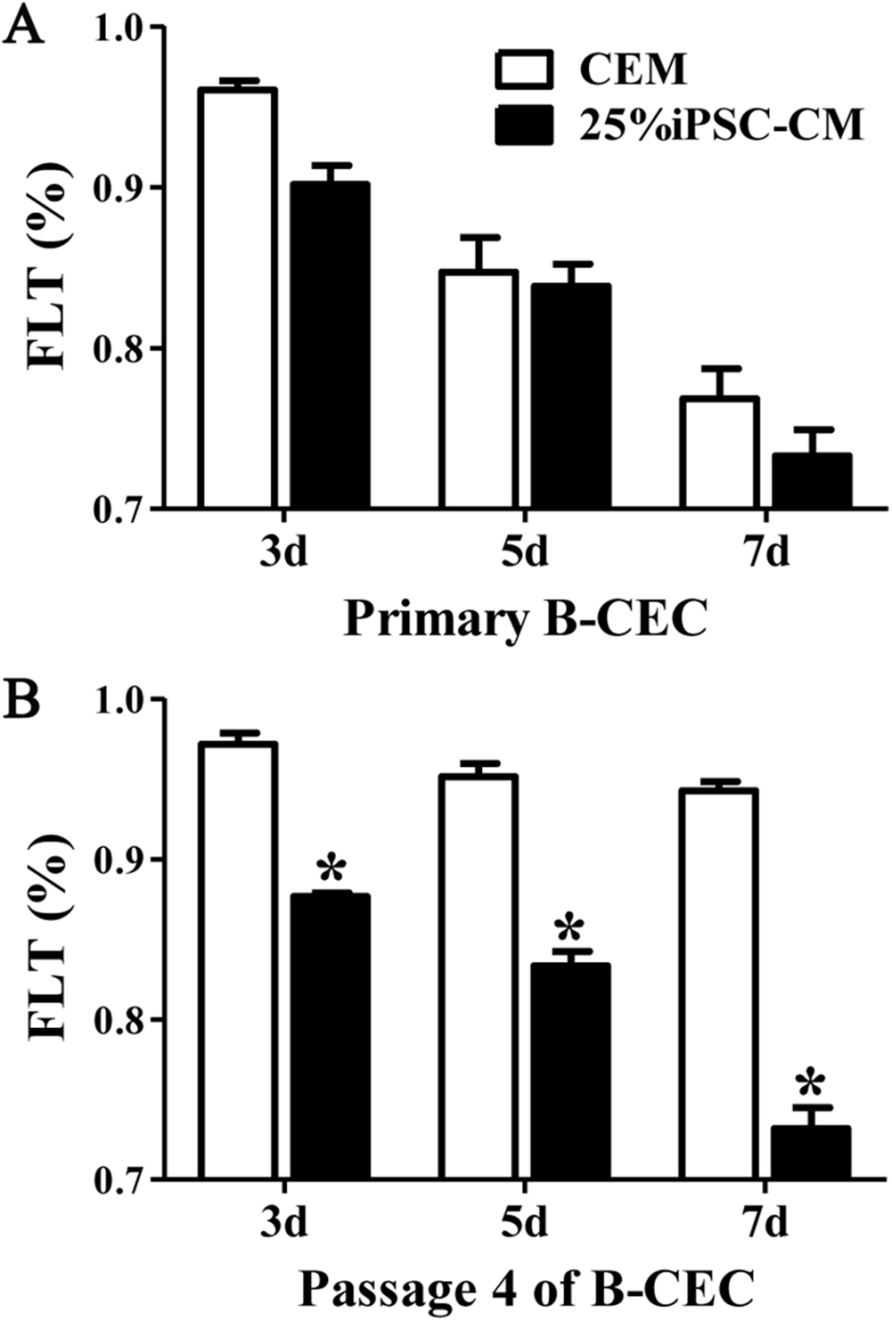
The percentage of fluorescein leakage was determined with ELISA. (A) Quantification of FLK assays at P0 showed no significant difference (*P* > 0.05, *n* = 3) between the 25% iPSC-CM group and CEM group (at 3, 5, and 7 days). (B) Quantification of FLK assays at P4 showed a significant difference (*P < 0.001 versus CEM group, *n* = 3) between the 25% iPSC-CM group and CEM group (at 3, 5, and 7 days).

The fluorescein leakage data showed that there were no significant differences between B-CECs at passage 0 in the iPSC-CM and CEM groups. The iPSC-CM group showed 0.90% ± 0.02% at 3 days, 0.83% ± 0.02% at 5 days, and 0.73% ± 0.03% at 7 days, while the CEM group showed 0.96% ± 0.01% at 3 days, 0.84% ± 0.04% at 5 days, and 0.76% ± 0.03% at 7 days (*P* > 0.05, *n* = 6) (Fig. 5A). However, there were significant differences of B-CECs between the iPSC-CM and CEM groups at passage 4. The iPSC-CM group showed 0.87% ± 0.01% at 3 days, 0.83% ± 0.02% at 5 days, and 0.73% ± 0.03% at 7 days, while the CEM group showed 0.97% ± 0.02% at 3 days, 0.95% ± 0.02% at 5 days, and 0.94% ± 0.01% at 7 days (*P* < 0.01, *n* = 6) (Fig. 5B). Therefore, we conclude that B-CECs at early passage cultured in iPSC-CM have barrier functions similar to those cultured in CEM, while they have a better barrier function after several passages than those cultured in CEM.

### The effect of PI3-kinase in iPSC-CM on promoting B-CEC proliferation

**Figure 6:**
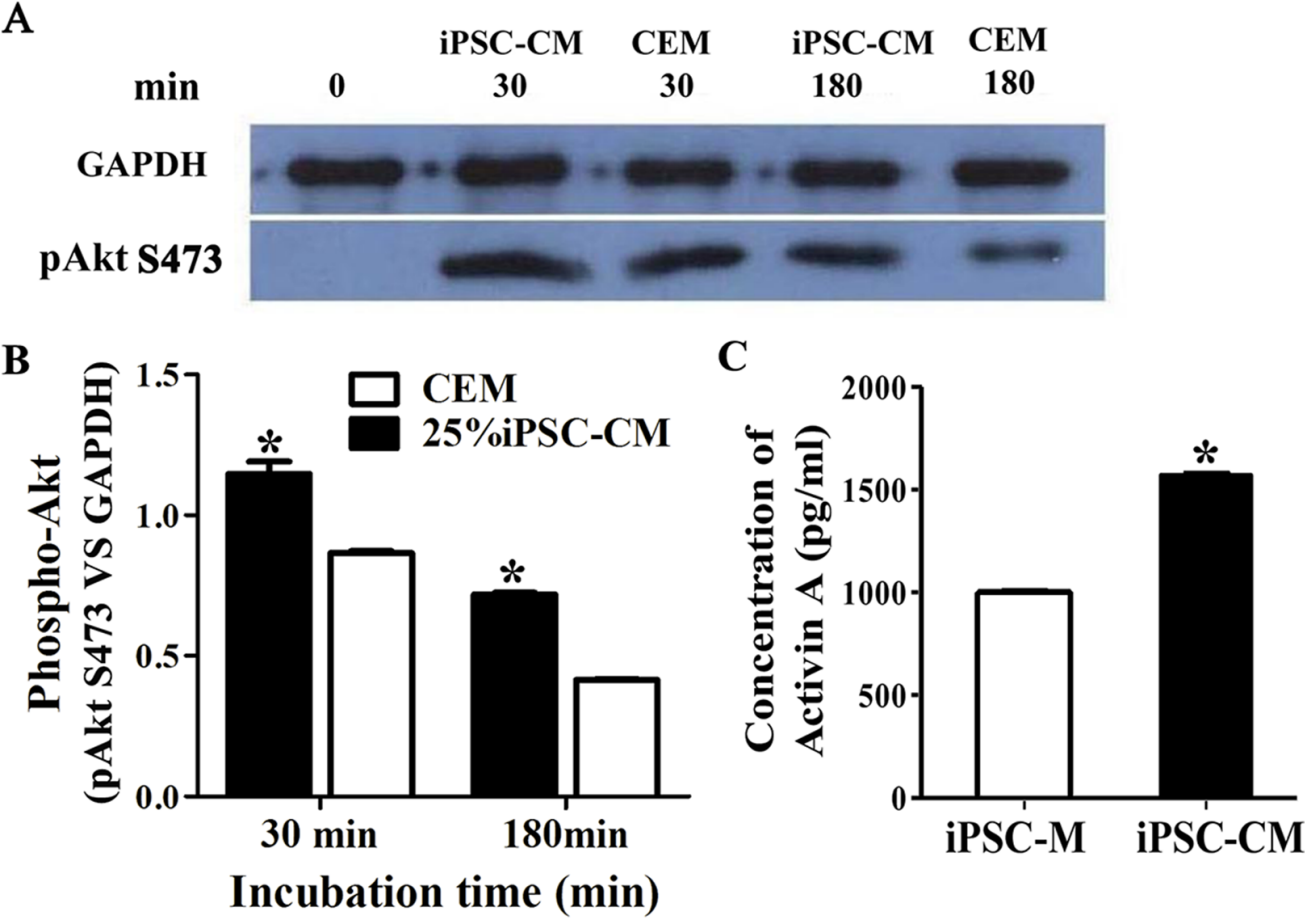
The effect of PI3-kinase in iPSC-CM on promoting B-CEC proliferation. (A) Western blotting showed the pAkt levels in the B-CECs cultured in either 25% iPSC-CM or CEM for 30 min and 180 min, after starvation treatment. (B) Quantification of the western blot assays showed that the 25% iPSC-CM group had higher pAkt expression than the control group (at 30 and 180 min), and the difference was statistically significant (*P < 0.05 versus CEM group, *n* = 3). (C) ELISA detection showed that activin A expression was significantly higher in iPSC-CM medium than in normal iPSC medium, and the difference was statistically significant (*P < 0.01 versus CEM group, *n* = 3).

The phosphorylation of Akt was evaluated by Western blot analysis when serum-starved B-CECs were treated with iPSC-CM or CEM for 30 or 180 min. The results revealed that phosphorylation of Akt in B-CECs treated with CEM was greatly induced at 30 min, while it attenuated in 180 min. On the other hand, phosphorylation of Akt in B-CECs treated with iPSC-CM showed sustained phosphorylation until 180 min, as shown in Fig. 6A. There were statistic differences between the two groups at 30 min and 180 min, as shown in Fig. 6B (*P* < 0.01, *n* = 3). These findings indicate that iPSC-CM may employ PI3-kinase signaling to regulate cell cycle progression and promote B-CEC proliferation.

### The effect of activin A on B-CEC growth

**Figure 7:**
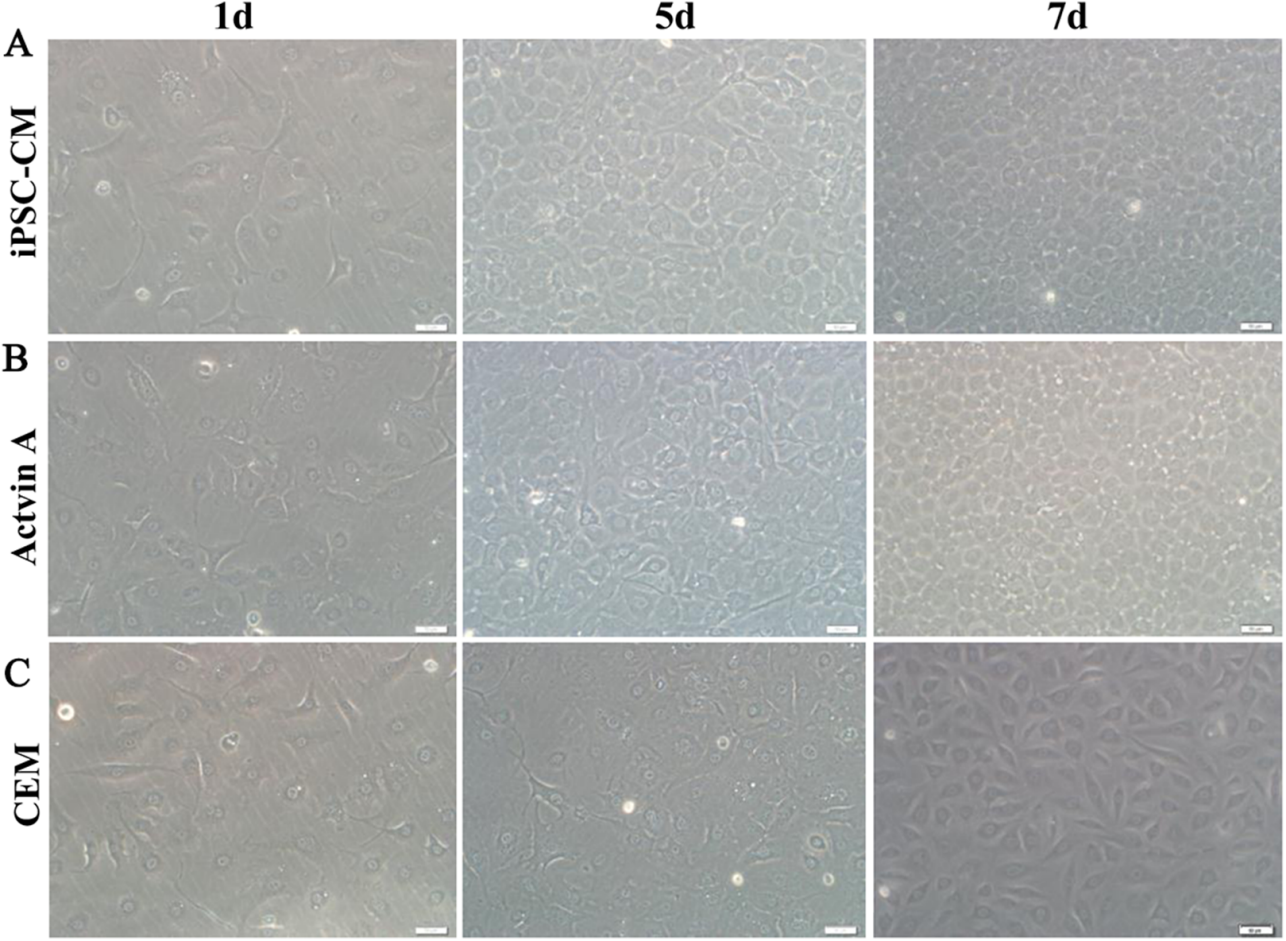
Morphology change of B-CECs with activin A. iPSC-CM (A) and activin A (B) can restore the hexagonal paving stone shape of B-CECs. The cell morphological diversity could not be restored to its typical form in the control group (C). Scale bar: 50 μm.

Here, we used ELISA to test the content of activin A in fresh iPSC medium and in iPSC-CM. As shown in Fig. 6C, we found that the concentration of activin A in iPSC-CM (1,594.76 ± 45.78 pg/ml) was significantly higher than that in fresh iPSC medium (1,011.26 ± 42.59 pg/ml) (*P* < 0.01, *n* = 3). B-CECs in CEM lost hexagonal morphology and became irregularly shaped, as shown in Figs. 7A–7C. However, the morphology of B-CECs in CEM supplemented with activin A retained a hexagon shape, and these cells looked similar to the B-CECs in iPSC-CM. These findings indicate that activin A is one of the important components of iPSC-CM, maintaining endothelial cell morphology after several passages.

## Discussion

In current study, we found that B-CECs showed higher gene expressions of CEC-related phenotypes (Na^+^-K^+^-ATPase, COL4A, and COL8A) and proliferation-related NIFK when cultured in iPSC-CM than those cultured in CEM. Our scratch test also demonstrated that iPSC-CM had a more positive effect on the B-CEC healing ability compared to CEM. Interestingly, our fluorescein leakage test also showed that B-CECs at P4 in iPSC-CM had a better barrier function than those in CEM, even though B-CECs at P0, both in iPSC-CM and CEM, had similar fluorescein leakage results. Correspondingly, B-CECs at P4 in iPSC-CM exhibited a smaller number of apoptotic and necrotic cells. Since specific markers of CECs have not yet been established, we selected markers that characterize corneal endothelial function. It is well known that CECs are rich in Na^+^/K^+^-ATPase and AQP1, which are associated with the pump functions of CECs (Thériault et al., 2018). NIFK gene encodes a protein that interacts with the fork head-associated domain of the Ki-67 antigen. The encoded protein may bind RNA and may play a role in mitosis and cell cycle progression (Byeon et al., 2005; Takagi et al., 2001). Ki67 is involved in the regulation of cell cycle progression, including DNA replication, regulation of chromosome configurator and interactions with actin in controlling centrosome separation (Scholzen & Gerdes, 2000). The lack of Ki67 gene expression is closely associated with abnormal cell cycle events, especially in the M phase. Petroll et al. (1999) have shown that Ki67 can be used to monitor the proliferation of corneal endothelial cells after scrape injury.

iPSCs can secrete cytokines, chemokines, growth factors, metabolites, and bioactive lipids. A wide range of products from iPSC secretion could encourage the growth of other cells (Lane, Williams & Watt, 2014). iPSC-CM has previously been shown to improve tissue repair in different disease models (Darabi et al., 2012; Mou et al., 2012; Oki et al., 2012). For example, in a mouse model of ventilator-induced lung injury (VILI), iPSC-CM suppressed high tidal volume-induced VILI, as observed by decreasing lung edema, microvascular permeability, neutrophil infiltration, and elevating the PaO2/FiO2 ratio in bronchial epithelium in response to these treatments (Li et al., 2013a). iPSC-CM could enhance alveolar epithelial regeneration in vivo partially due to containing hepatocyte growth factor (Gazdhar et al., 2014). iPS-CM could reduce apoptosis, oxidative stress, and fibrosis, as well as improve cardiac function in diabetic rat models (Neel & Singla, 2011). In this study, we found that compared with CEM, iPSC-CM not only increased B-CEC proliferation but also promoted better contact-inhibited monolayer shape. The percentage of B-CECs entering S and G2 phase when treated with iPSC-CM was also greater than those cultured in CEM.

The mechanism of the effects of iPSC-CM on B-CECs was preliminarily investigated. We starved the cells before cultivation with iPSC-CM or CEM to ensure that a large group of cells would enter the proliferation stage from the stationary phase and then checked the phosphorylated Akt expression. B-CEC expression of phosphorylated Akt increased significantly after incubating in 25% iPSC-CM medium or CEM medium for 30 min. However, phosphorylated Akt was significantly decreased in cells incubated in CEM after 180 min, while it remained high in the iPSC-CM cells. Our results showed that 25% iPSC-CM promoted the proliferation of B-CECs via an effect on the PI3-kinase signaling pathway leading to sustained activation due to the formation of a cascade signal amplification for cell proliferation. Nakahara et al. (2013) reported that the proliferative effect of bone marrow MSC-CM was facilitated via the downregulation of p27 and the upregulation of cyclin D through phosphatidylinositol three-kinase (PI3-kinase). Phosphorylation of Akt and ERK1/2 was observed in HCECs after exposure to MSC-CM.

There are a variety of soluble paracrine factors released from pluripotent stem cells (Zhang et al., 2014). Activin A is a member of the transforming growth factor-β superfamily; it harbors a multitude of functions such as regulating a broad range of cellular processes, including proliferation, differentiation, and apoptosis (Licona-Limón et al., 2009). Activin A is a key regulator in the maintenance of self-renewal and pluripotent status and supports feeder cells and serum-free growth in iPS and embryonic stem (ES) cell cultures (Beattie et al., 2005). Human ES cells rely on activin A to regulate the expression of nanog (James et al., 2005; Shin et al., 2011). Activin A is important for the survival and growth of single iPS cells. Our previous study showed that the enhanced growth of single iPS cells based on the conditioned medium culture of half-exchange mTeSR1 medium (HM) and iPS-CM was associated with increased bFGF and activin A (Guo et al., 2015). We also found that the promoting effects of iPS-CM on the proliferation and anti-apoptosis of human adipose derived stem cells might be partly associated with increased activin A levels in the supernatant (Lian et al., 2016). In this study, we found that activin A levels in iPSC-CM increased significantly. B-CECs at P4 in CEM lost hexagonal shape and became irregularly shaped. However, the morphology of B-CECs in CEM supplemented with activin A recovered their hexagonal shape like the B-CECs in iPSC-CM. Therefore, activin A is one of the effective components of iPSC-CM that promotes B-CEC activity, and it also plays a key role in maintaining cell morphology.

In this study, the species mismatch limits the translation of the presented basic research to clinical application. However, the species mismatch was chosen to be able to discriminate between the role of the different cell types (Pleumeekers et al., 2018). The limited proliferative ability of HCECs makes the in vitro propagation of CECs difficult, while B-CECs are relatively easy to obtain. Cultured B-CECs provide us with an excellent in vitro model to study the differentiated functions of the corneal endothelium (Savion et al., 1982). B-CECs have been shown to remain functional in vitro and can replace a damaged endothelium in vivo (Gospodarowicz, Greenburg & Alvarado, 1979a, 1979b). Therefore, investigations and observations of cellular behaviors in the B-CEC model can also be informative for human CEC biology and applications. As a step toward clinical applications, a further study on HCECs will next be undertaken.

## Conclusion

In summary, this study is the first to demonstrate the beneficial effects of iPSC-CM on the improved growth of B-CECs, which was partly related to the elevated levels of activin A in the supernatant. Similar morphological changes were observed in cells grown in iPSC-CM and in cells grown in CEM with activin A supplements. This proves that activin-A plays an important role in maintaining CEC morphology. Our findings indicated that iPSC-CM stimulated the proliferation of B-CECs not only by regulating the G2 and S phase in the cell cycle and increasing the expression of proliferation-related gene NIFK but also by reducing apoptosis and maintaining barrier and secretion functions after several passages. The enhanced properties were achieved by adjusting the expression of phosphorylation of Akt through the PI3-kinase pathway, which suggested that B-CECs maintained in iPSC-CM acquired the corneal endothelium stem-cell-like property. This study provides the basis for the possibility to expand HCECs, which could contribute to the treatment of endothelial dysfunctions. More research is required to further explore and verify the active components of iPSC-CM.

## Supplemental Information

10.7717/peerj.6734/supp-1 Supplemental Information 1 Raw data for WB blotting.

10.7717/peerj.6734/supp-2 Supplemental Information 2 Raw data used for plots, error bars, and statistical analysis.

## Additional Information and Declarations

### Data Availability

The following information was supplied regarding data availability: The raw measurements are available in the Supplementary Files.
