# Supplementary figures and images for: Characterization of the iPSC-derived conditioned medium that promotes the growth of bovine corneal endothelial cells
Source: PeerJ. 2019 Apr 16;7:e6734. doi: 10.7717/peerj.6734 (PMC6474332; doi:10.7717/peerj.6734)

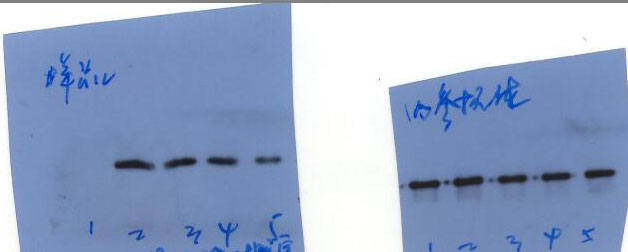


PAkt-S473

S473

GAPDH

S473

Supplement: Supplemental Information 1 [file peerj-07-6734-s001.docx]
